# Supplementary material for: Demographic, social and health system factors associated with maternal mortality in Pakistan: A nested case-control study
Source: PLoS One. 2025 Jan 3;20(1):e0290492. doi: 10.1371/journal.pone.0290492 (PMC11698442; doi:10.1371/journal.pone.0290492)
Supplement: Attachment — Submitted filename: 2023 comment Pone-D-23-22204.docx [file pone.0290492.s001.docx]

To be accepted for publication in PLOS ONE, research articles must satisfy the following criteria:

1. The study presents the results of original research.

I am wondering this manuscript. I found publish in the medRxiv preprint doi: https://doi.org/10.1101/2023.08.10.23293928; this version posted August 15, 2023.

2. Results reported have not been published elsewhere.

I am wondering this manuscript. I found publish in the medRxiv preprint doi: https://doi.org/10.1101/2023.08.10.23293928; this version posted August 15, 2023.

3. Experiments, statistics, and other analyses are performed to a high technical standard and are described in sufficient detail.

No comment

4. Conclusions are presented in an appropriate fashion and are supported by the data.

No comment

5. The article is presented in an intelligible fashion and is written in standard English.

Yes it is “The article is presented in an intelligible fashion and is written in standard English”

6. The research meets all applicable standards for the ethics of experimentation and research integrity.

The ethics of experimentation and research integrity issue did not show in manuscript. If the project used the secondary data, IRB should be explained about exempt.

7. The article adheres to appropriate reporting guidelines and community standards for data availability.

No comment
