# Supplementary material for: Demographic, social and health system factors associated with maternal mortality in Pakistan: A nested case-control study
Source: PLoS One. 2025 Jan 3;20(1):e0290492. doi: 10.1371/journal.pone.0290492 (PMC11698442; doi:10.1371/journal.pone.0290492)
Supplement: Attachment — Submitted filename: Why Mothers Continue To Die In Pakistan.docx [file pone.0290492.s002.docx]

**Why Mothers Continue To Die In Pakistan: A Nested Case-Control Study Of Predictors Of Maternal Mortality**

**Summary of the Research**

Maternal mortality ratio (MMR) declined in Pakistan from 276 maternal deaths per 100,000 live births in 2006-07 to 186 in 2019. Despite this decline, reasons why mothers continue to die, the inequity in the burden of maternal mortality and its predictors largely remain unknown. The in-depth analysis of Pakistan Maternal Mortality Survey 2019 was undertaken using the nested case-control design. Cases and controls were similar on access to antenatal care (ANC) and ANC provider, but differed on age, education, number of pregnancies, type of delivery, tetanus toxoid vaccination during last pregnancy, and ever using a contraceptive method. More of the cases had their last delivery by a skilled birth attendant (83% compared to 63% among controls) and delivered at the government hospital (43% compared to 33% among controls) while home delivery was relatively more common among controls (32% compared to 25% among cases). Odds of maternal death were lowest for women giving birth during age 20-29 (odds ratio – OR: 0.5; 95% CI 0.23-1.07) as compared to women in age 15-19 and those age 30-39 (OR: 1.34; 95% CI 0.61-2.95) and 40-49 (OR: 1.21; 95% CI 0.42-3.45), and among women with secondary or higher education (OR: 0.35; 95% CI 0.17-0.74) compared to women with no education. Women in certain sub-groups confront higher risks of maternal death. Increasing female education, preventing early and late childbearing through contraceptive use, increasing tetanus vaccination during pregnancy, improving providers’ skills, and quality of health care are required to eliminate preventable maternal deaths in Pakistan.

**Areas for improvement**

**Title:**

Tile is concise and good well expressing and reflecting the content of the study.

**Abstract:**

The abstract is well written, however, the authors should revise the language to improve readability.

The authors should make sure that the abstract don’t exceed 300 words.

**Introduction:**

The authors should write details in introduction section to tell the reader about the general information about the phenomena under study.

The authors should mention the significant of the study and identify the gap in knowledge as well.

The authors should revise introduction section for grammar issues and language to improve readability.

The authors should clear identify the variables of the study.

The authors should explain theoretical framework of the study.

The authors should mention the significance of the study.

The authors should make this section more clear, so readers will understand what message you wanted them to understand.

**Overall**

Good and brief introduction section.

**Material and Methods:**

Clear section but The authors should revise the language to improve readability.

The authors should mention why they selected the design of the study clearly.

The authors should briefly explain what is PMMS and provide the full for of this abbreviation. From where they got this questionnaire.

The authors should mention about verbal autopsy (VA) questionnaire, is it different from PMMS questionnaire.

The authors should mention about other ethical consideration for data control.

The authors should briefly explain about Stata version 14.

I am not sure if there are specific criteria to include the participants, mention that please!

**Results, discussion and conclusion:**

Clear results, discussion and conclusion sections. But improve grammar and language for readability.

**References:**

The authors should revise all references according to the guidelines provided.

Make sure that all intext referencing reflecting with in the references list.
