# Supplementary material for: Demographic, social and health system factors associated with maternal mortality in Pakistan: A nested case-control study
Source: PLoS One. 2025 Jan 3;20(1):e0290492. doi: 10.1371/journal.pone.0290492 (PMC11698442; doi:10.1371/journal.pone.0290492)
Supplement: Attachment — Submitted filename: Response to Reviewers.docx [file pone.0290492.s003.docx]

Response to Reviewers

Reviewer #1: 1. The study presents the results of original research.
I am wondering this manuscript. I found publish in the medRxiv preprint doi: <https://doi.org/10.1101/2023.08.10.23293928>; this version posted August 15, 2023.
2. Results reported have not been published elsewhere.
I am wondering this manuscript. I found publish in the medRxiv preprint doi: <https://doi.org/10.1101/2023.08.10.23293928>; this version posted August 15, 2023.

***Response: Thank you for pointing this out. We initially submitted the manuscript to BMG Global Health that suggested a referral to another journal, but we declined and submitted the manuscript only to PlosOne, after some revisions. We did not submit the manuscript to medRxiv or to any other journal and are surprised to find it on medRxiv as a “preprint” DOI. We have no idea how medRxiv found and placed the manuscript and referred it as “preprint” version when it has not yet been accepted by PlosOne. We confirm that the manuscript has not been published nor submitted to any other journal. Following the reviewer’s comment, we browsed the DOI version and find it different from the revised one.***

3. Experiments, statistics, and other analyses are performed to a high technical standard and are described in sufficient detail.
No comment
4. Conclusions are presented in an appropriate fashion and are supported by the data.
No comment
5. The article is presented in an intelligible fashion and is written in standard English.
Yes it is “The article is presented in an intelligible fashion and is written in standard English”
6. The research meets all applicable standards for the ethics of experimentation and research integrity.
The ethics of experimentation and research integrity issue did not show in manuscript. If the project used the secondary data, IRB should be explained about exempt.

***Response: The manuscript used publically available data of Pakistan Maternal Mortality Survey (PMMS). The authors had no role in data collection that was undertaken by National Institute of Population Studies (NIPS) after obtaining the necessary ethical approvals and complying with ethical requirements of informed consent, privacy and confidentiality. We have added information under Ethical Statement. The PMMS data are available for download from:*** [***https://dhsprogram.com/methodology/survey/survey-display-552.cfm***](https://dhsprogram.com/methodology/survey/survey-display-552.cfm)***.***
7. The article adheres to appropriate reporting guidelines and community standards for data availability.
No comment

Reviewer #2: Why Mothers Continue To Die In Pakistan: A Nested Case-Control Study Of Predictors Of Maternal Mortality

Summary of the Research
Maternal mortality ratio (MMR) declined in Pakistan from 276 maternal deaths per 100,000 live births in 2006-07 to 186 in 2019. Despite this decline, reasons why mothers continue to die, the inequity in the burden of maternal mortality and its predictors largely remain unknown. The in-depth analysis of Pakistan Maternal Mortality Survey 2019 was undertaken using the nested case-control design. Cases and controls were similar on access to antenatal care (ANC) and ANC provider, but differed on age, education, number of pregnancies, type of delivery, tetanus toxoid vaccination during last pregnancy, and ever using a contraceptive method. More of the cases had their last delivery by a skilled birth attendant (83% compared to 63% among controls) and delivered at the government hospital (43% compared to 33% among controls) while home delivery was relatively more common among controls (32% compared to 25% among cases). Odds of maternal death were lowest for women giving birth during age 20-29 (odds ratio – OR: 0.5; 95% CI 0.23-1.07) as compared to women in age 15-19 and those age 30-39 (OR: 1.34; 95% CI 0.61-2.95) and 40-49 (OR: 1.21; 95% CI 0.42-3.45), and among women with secondary or higher education (OR: 0.35; 95% CI 0.17-0.74) compared to women with no education. Women in certain sub-groups confront higher risks of maternal death. Increasing female education, preventing early and late childbearing through contraceptive use, increasing tetanus vaccination during pregnancy, improving providers’ skills, and quality of health care are required to eliminate preventable maternal deaths in Pakistan.
Areas for improvement
Title:
Tile is concise and good well expressing and reflecting the content of the study.

***Response: Thank you. Reviewer #3 suggested a different title and we have followed the suggestion and changed it to “Demographic, social and health system factors associated with maternal mortality in Pakistan: A nested case-control study”***
Abstract:
The abstract is well written, however, the authors should revise the language to improve readability.
The authors should make sure that the abstract don’t exceed 300 words.
***Response: Thank you for the suggestion. We have revised the abstract to simplify and improve the readability. The word count is now 263.***

Introduction:
The authors should write details in introduction section to tell the reader about the general information about the phenomena under study.

***Response: We have further revised the Introduction and summarized the general information available on maternal mortality in Pakistan.***
The authors should mention the significant of the study and identify the gap in knowledge as well.

***Response: Thank you for the suggestion. We have added: “However, there remains a dearth of understanding regarding the contextual, health system, social, and biological factors that significantly influence the reduction of preventable maternal deaths in Pakistan. By identifying subgroups of women with high risk of maternal mortality, this study aims to call for concerted efforts focusing on these groups. The study also aims to pinpoint interventions that are needed to reduce preventable maternal deaths in Pakistan. In addition, it highlights the application of case-control methodology to the analysis of maternal mortality data collected in cross-sectional surveys such as Demographic and Health Surveys and Maternal Mortality Surveys.”***
The authors should revise introduction section for grammar issues and language to improve readability.

***Response: We have revised the text in Introduction to address the comment.***
The authors should clear identify the variables of the study.
The authors should explain theoretical framework of the study.
The authors should mention the significance of the study.
The authors should make this section more clear, so readers will understand what message you wanted them to understand.

***Response: Thank you for the above suggestions. We have addressed these in our revisions. The first two suggestions have been addressed under Statistical Analysis section where we have added the following:***

***“Operationalizing the conceptual framework shown in Figure 1, maternal death was taken as an outcome variable that was dichotomized into whether the death occurred or not (i.e., cases were maternal deaths, and controls were women who were alive and had a live birth). The proximate determinants of the outcome of maternal death are the health seeking behaviors such as seeking antenatal care (ANC); antenatal care provider; month of pregnancy at first ANC visit; receiving tetanus toxoid (TT) injection during pregnancies and comorbidities such as composite index of complications and composite symptoms. Socio-economic variables examined for their potential role in women level risk factors and health-seeking behaviors to ultimately exacerbate or reduce the risk of maternal death are region and urban/rural place of residence and educational level of women. Women level risk factors include age; parity; number of living children, ever had caesarean section; place of delivery; assistance with delivery; type of delivery and contraceptive usage. Together, these variables attenuate or escalate the risk of maternal mortality.”***

***Suggestion #3 and 4 are addressed in the revision of Introduction as also explained above.***
Overall
Good and brief introduction section.
Material and Methods:
Clear section but The authors should revise the language to improve readability.

***Response: We have revised to simplify and improve readability.***
The authors should mention why they selected the design of the study clearly.

***Response: We have expanded the text in Materials and Methods to provide the requested information as follows:***

***PMMS and other cross-sectional surveys covering maternal deaths often use verbal autopsy module to ascertain information about the deceased. Case-control design is an appropriate option to study the outcome of maternal mortality where those who died are classified as “cases” and those who survived as “controls”. The purpose of classifying the two groups on outcome, that is, maternal death in this study, is to investigate the underlying factors that distinguish the two groups.***

The authors should briefly explain what is PMMS and provide the full for of this abbreviation.

***Response: Information on PMMS is provided under Materials and Methods. The abbreviation of PMMS is defined when first used in the Abstract and in Introduction and also in the text.***

From where they got this questionnaire.

***Response: The survey was conducted by the National Institute of Population Studies (NIPS) under the auspices of the Demographic and Health Survey Program. The final report of the survey with all details on sampling procedures and the questionnaires as well as data are available at:*** [***https://dhsprogram.com/methodology/survey/survey-display-552.cfm***](https://dhsprogram.com/methodology/survey/survey-display-552.cfm)***.***
The authors should mention about verbal autopsy (VA) questionnaire, is it different from PMMS questionnaire.

***Response: PMMS questionnaire included verbal autopsy module. Information is available at:*** [***https://dhsprogram.com/methodology/survey/survey-display-552.cfm***](https://dhsprogram.com/methodology/survey/survey-display-552.cfm)***.***
The authors should mention about other ethical consideration for data control.

***Response: We have expanded information under Ethical Statement.***
The authors should briefly explain about Stata version 14.

***Response: We have added the information.***
I am not sure if there are specific criteria to include the participants, mention that please!

***Response: The criteria for the participation of respondents in PMMS is described in the final report of PMMS. See*** [***https://dhsprogram.com/publications/publication-FR366-Other-Final-Reports.cfm***](https://dhsprogram.com/publications/publication-FR366-Other-Final-Reports.cfm) ***and for the study mentioned under Materials and Methods and shown in Figure 2.***
Results, discussion and conclusion:
Clear results, discussion and conclusion sections. But improve grammar and language for readability.

***Response: We have revised to improve readability.***
References:
The authors should revise all references according to the guidelines provided.
Make sure that all intext referencing reflecting with in the references list.

***Response: Thank you for suggestion, we have revised the references according to the journal referencing guidelines.***

Reviewer #3: Well done for the reviewers in working with two databases to address an important global health problems. However, there are still some areas that need major revisions. Please see comments below:

Title: I feel like the title is too broad, at first sight I though this paper included demographic, social, economic, cultural factors associated with maternal mortality in Pakistan. I suggest using something more succinct like, "Demographic, Social and Clinical Factors associated with maternal mortality in Pakistan: A nested case control study"

***Response: Thank you for suggesting the alternative title. We have gratefully accepted your suggestion with a minor change from “clinical” to “health system”.***

Abstract: The abstract needs a lot of work, see couple suggestions below;

Background: In Parkistan, there has been a decline of maternal mortality by x% between 2006-2019. However, the MMR remains relatively high at xxxxx. This study aims to assess factors associated with MMR in Pakistan.

Methods: We used data from PMMS 2019. We employed a nested case control design. Cases were defined as.......Controls were defined as. We performed a multivariate regression with the following independent variable. The dependent variable is maternal mortality.

Results: Results are currently overwhelming. consider picking 2-3 from table one. And presenting odds rations for statistically significant predictors and surprising findings.

***Response: Thank you for the suggestions. We have revised the Abstract incorporating your suggestions.***

Introduction: First paragraph could be more succinct and shorter focusing mostly on Pakistan. Currently too wordy. Second paragraph needs to be elaborated more. We need more information on the conceptual framework (what informed it? any established theories, key associations from the prior studies mentioned in the past? Do you have a DAG? Rationale and objective of this study needs to be clearly linked.
Figure 1 is nice clear diagram

***Response: Thank you. We have revised the Introduction and have added information on conceptual framework under Statistical Analysis on the linkages among variables identified in the framework.***

***We had a TAC (Technical Advisory Committee) that reviewed the study design, analysis and the results.***

Methods: Methods section looks generally good. However see comments in abstract.
Did you perform a multistage cluster design or you performed a secondary data analysis from the survey using a nested control study design?

***Response: We performed secondary data analysis of the survey by defining cases and controls as part of the analysis of data.***

is there room for Flexibility in a statistical analysis plan. Won't this introduce Bias (line 111-114)

***Response: By “flexibility”, we implied the ability to conduct analysis of “observational” data with subgroups that experience the event, that is, maternal death, and those who did not and to identify factors associated with the outcome. This does not introduce bias.***

Figure 2: I am not an expert in matching cases and controls so I stand to be corrected. What is the rationale of matching cases and controls by clusters? What is the matching ratio 1 case to 5 controls or 1 case to 4 controls. What exactly is happening here? And is it statistically correct?

***Response: We matched cases and controls to have both from the same region and place of residence and thus comparable on this aspect. The ratio of 1 to 4 or 1 to 5 is statistically valid. Of course, a ratio of 1 to 1 is better, but maternal death are (fortunately) less than those who gave birth and were alive. The ratio of 1 case to 5 controls provide statistically valid results.***

Results: Improve structure and clarity in results section. Try to use sub-titles. See also comments in abstract. Read how other articles presents such data.

***Response: Thank you for the helpful suggestions. We have summarized results under sub-titles of: (1) Profile of cases and controls and risk factors; and (2) predictors of maternal mortality***
Discussion: Consider formatting it in this way.

1. Key findings, " We assessed the socio-demographic and clinical factors associated with maternal mortality in Pakistan. We found that xxxxxxxxx

2. Similarities or lack of with existing literature on the topic

3. Limitations

4. Recommendations

***Response: Thank you. We have revised Discussions to incorporate suggestions.***

6. PLOS authors have the option to publish the peer review history of their article ([what does this mean?](https://journals.plos.org/plosone/s/editorial-and-peer-review-process#loc-peer-review-history)). If published, this will include your full peer review and any attached files.


If you choose “no”, your identity will remain anonymous but your review may still be made public.


**Do you want your identity to be public for this peer review?** For information about this choice, including consent withdrawal, please see our [Privacy Policy](https://www.plos.org/privacy-policy).

Reviewer #1: No

Reviewer #2: **Yes:** Zalikha Khamis Al-Marzouqi

Reviewer #3: No
