# Supplementary material for: Demographic, social and health system factors associated with maternal mortality in Pakistan: A nested case-control study
Source: PLoS One. 2025 Jan 3;20(1):e0290492. doi: 10.1371/journal.pone.0290492 (PMC11698442; doi:10.1371/journal.pone.0290492)
Supplement: Attachment — Submitted filename: Response to Reviewers_July 2024.docx [file pone.0290492.s004.docx]

**From:** "PLOS ONE" <[em@editorialmanager.com](mailto:em@editorialmanager.com)>

**To:** "Ahsan Maqbool Ahmad" <[drahsen@yahoo.com](mailto:drahsen@yahoo.com)>

**Cc:**

**Sent:** Mon, 17 Jun 2024 at 3:21 am

**Subject:** PLOS ONE Decision: Revision required [PONE-D-23-22204R1] - [EMID:59c3215e5b560e48]

PONE-D-23-22204R1

Demographic, social and health system factors associated with maternal mortality in Pakistan: A nested case-control study

PLOS ONE

Dear Dr. Maqbool Ahmad,

Thank you for submitting your manuscript to PLOS ONE. After careful consideration, we feel that it has merit but does not fully meet PLOS ONE’s publication criteria as it currently stands. Therefore, we invite you to submit a revised version of the manuscript that addresses the points raised during the review process.

Please submit your revised manuscript by Jul 31 2024 11:59PM. If you will need more time than this to complete your revisions, please reply to this message or contact the journal office at [plosone@plos.org](mailto:plosone@plos.org). When you're ready to submit your revision, log on to <https://www.editorialmanager.com/pone/> and select the 'Submissions Needing Revision' folder to locate your manuscript file.

Please include the following items when submitting your revised manuscript:

- A rebuttal letter that responds to each point raised by the academic editor and reviewer(s). You should upload this letter as a separate file labeled 'Response to Reviewers'.
- A marked-up copy of your manuscript that highlights changes made to the original version. You should upload this as a separate file labeled 'Revised Manuscript with Track Changes'.
- An unmarked version of your revised paper without tracked changes. You should upload this as a separate file labeled 'Manuscript'.

If you would like to make changes to your financial disclosure, please include your updated statement in your cover letter. Guidelines for resubmitting your figure files are available below the reviewer comments at the end of this letter.

If applicable, we recommend that you deposit your laboratory protocols in [protocols.io](http://protocols.io) to enhance the reproducibility of your results. Protocols.io assigns your protocol its own identifier (DOI) so that it can be cited independently in the future. For instructions see: <https://journals.plos.org/plosone/s/submission-guidelines#loc-laboratory-protocols>. Additionally, PLOS ONE offers an option for publishing peer-reviewed Lab Protocol articles, which describe protocols hosted on [protocols.io](http://protocols.io). Read more information on sharing protocols at <https://plos.org/protocols?utm_medium=editorial-email&utm_source=authorletters&utm_campaign=protocols>.

We look forward to receiving your revised manuscript.

Kind regards,

Abera Mersha, MSc.
Academic Editor
PLOS ONE


[Note: HTML markup is below. Please do not edit.]

Reviewers' comments:

Reviewer's Responses to Questions

**Comments to the Author**

1. If the authors have adequately addressed your comments raised in a previous round of review and you feel that this manuscript is now acceptable for publication, you may indicate that here to bypass the “Comments to the Author” section, enter your conflict of interest statement in the “Confidential to Editor” section, and submit your "Accept" recommendation.

Reviewer #2: All comments have been addressed

Reviewer #3: All comments have been addressed

2. Is the manuscript technically sound, and do the data support the conclusions?

The manuscript must describe a technically sound piece of scientific research with data that supports the conclusions. Experiments must have been conducted rigorously, with appropriate controls, replication, and sample sizes. The conclusions must be drawn appropriately based on the data presented.

Reviewer #2: Yes

Reviewer #3: Partly

***Response: We have revised the text in line with the comments and suggestions received.***

3. Has the statistical analysis been performed appropriately and rigorously?

Reviewer #2: Yes

Reviewer #3: Yes

4. Have the authors made all data underlying the findings in their manuscript fully available?

The [PLOS Data policy](http://www.plosone.org/static/policies.action#sharing) requires authors to make all data underlying the findings described in their manuscript fully available without restriction, with rare exception (please refer to the Data Availability Statement in the manuscript PDF file). The data should be provided as part of the manuscript or its supporting information, or deposited to a public repository. For example, in addition to summary statistics, the data points behind means, medians and variance measures should be available. If there are restrictions on publicly sharing data—e.g. participant privacy or use of data from a third party—those must be specified.

Reviewer #2: Yes

Reviewer #3: No

***Response: Pakistan Maternal Mortality Survey 2019 data are in public domain and can be downloaded from the website:*** [***https://dhsprogram.com/data/available-datasets.cfm***](https://dhsprogram.com/data/available-datasets.cfm)***. The survey was conducted by the National Institute of Population Studies.***

5. Is the manuscript presented in an intelligible fashion and written in standard English?

PLOS ONE does not copyedit accepted manuscripts, so the language in submitted articles must be clear, correct, and unambiguous. Any typographical or grammatical errors should be corrected at revision, so please note any specific errors here.

Reviewer #2: Yes

Reviewer #3: Yes

6. Review Comments to the Author

Please use the space provided to explain your answers to the questions above. You may also include additional comments for the author, including concerns about dual publication, research ethics, or publication ethics. (Please upload your review as an attachment if it exceeds 20,000 characters)

Reviewer #2: Well done team, all the best.

The authors addressed all changes

No more comments for them.

I wish them all the best

Reviewer #3: Abstract

This conclusion is off – how do you make such a conclusion from these results that are only looking at association and not causality?
I would suggest_
“Conclusion: This study provides factors associated with maternal mortality. Further investigation is needed on the determinants positively associated with high mortality rates.”

There is a limitation on what you can say when assessing causality or maybe you can argue that maternal deaths are rare and hence odds ratio = relative risk ratio, read up on it.

***Response: Thank you. We agree with the comment and suggestion and have revised the text to:*** ***“This study identifies factors associated with maternal mortality in Pakistan. Further investigation is needed on the determinants of high maternal mortality****.****”***

Introduction

Improved – However I would remove sections that speaks about the methodology in the introduction

There is a specific section for methodology

***Response: Thank you for the helpful suggestion. We have moved the relevant text to Methods section.***

Methodology

Please follow the following format for clarity:

Design: Case-control design of PMMS and Surveys with Maternal Mortality data (Include years)
Setting and Population
Data Collection
Study Variable – including Primary Outcome
Type of analysis and Software
Ethical Considerations

***Response: We have followed the suggested format and reorganized the text into the suggested sub-sections.***

Figure 2: Scheme of cases and controls identification and matching is a great diagram, give the explanation you gave on the rationale of different matching ratios

***Response: Added in the text of manuscript as follows (row 145-149)***

*There were a total of 147 maternal deaths (eligible cases) in the PMMS 2019 survey. To maximize the statistical power of analysis, inclusivity for statistical analysis purposes was considered in a way, so that the information on maximum number of cases could be utilized. In this milieu, there were 136 cases were included in the final analysis on the basis that they had at-least one cluster matched control available. As a result, there were only four clusters for whom lesser number of controls per case (i.e. less than ratio of 1:5) were available*.

Results

“Multi-stage analyses included the phases of descriptive analysis and inferential analyses by applying
173 univariate and multivariate regression techniques. The proportional representation of cases versus controls
174 was similar for the matching variables of region/province of residence and place of residence (i.e. urban vs
175 rural).” - Belongs in the methodology section.

Begin like – Table shows the Number and percentage distribution (%) of cases and controls by background variables, Pakistan, 2019…………

***Response: Thank you for the helpful suggestions. We have moved the text, as suggested to Methodology section.***

Discussion

The discussion needs restructuring.

We assessed the socio-demographic and clinical factors associated with maternal mortality in Pakistan. We found that We Cases and controls were similar on access to antenatal care (ANC) and ANC provider but differed on age, education, number of pregnancies, type of delivery, tetanus toxoid vaccination during last pregnancy, and contraceptive usage. A higher proportion of cases had deliveries by skilled birth attendants (83% compared to 63% among controls) while home deliveries were more common among controls (32% compared to 25% among cases). Odds of maternal death were lowest among women aged 20-29 years (odds ratio – OR: 0.5; 95% CI 0.23-1.07) and those with secondary or higher education (OR: 0.35; 95% CI 0.17-0.74). Surprisingly, deliveries attended by skilled birth attendants were associated with higher odds of maternal death (OR: 4.07; 95% CI 2.19-7.57) compared to those who were not.

Second paragraph should speak of similarities or lack of with existing literature on the topic
Third paragraph should speak of of limitations which you already have.

Fourth paragraph should speak of Recommendations (please see comments in abstract.

***Response: We have restructured the Discussion and followed these helpful recommendations.***

7. PLOS authors have the option to publish the peer review history of their article ([what does this mean?](https://journals.plos.org/plosone/s/editorial-and-peer-review-process#loc-peer-review-history)). If published, this will include your full peer review and any attached files.

If you choose “no”, your identity will remain anonymous but your review may still be made public.

**Do you want your identity to be public for this peer review?** For information about this choice, including consent withdrawal, please see our [Privacy Policy](https://www.plos.org/privacy-policy).

Reviewer #2: **Yes:** Zalikha Khamis Al-Marzouqi

Reviewer #3: No

[NOTE: If reviewer comments were submitted as an attachment file, they will be attached to this email and accessible via the submission site. Please log into your account, locate the manuscript record, and check for the action link "View Attachments". If this link does not appear, there are no attachment files.]

While revising your submission, please upload your figure files to the Preflight Analysis and Conversion Engine (PACE) digital diagnostic tool, <https://pacev2.apexcovantage.com/>. PACE helps ensure that figures meet PLOS requirements. To use PACE, you must first register as a user. Registration is free. Then, login and navigate to the UPLOAD tab, where you will find detailed instructions on how to use the tool. If you encounter any issues or have any questions when using PACE, please email PLOS at [figures@plos.org](mailto:figures@plos.org). Please note that Supporting Information files do not need this step.

*In compliance with data protection regulations, you may request that we remove your personal registration details at any time.* [*(Remove my information/details)*](https://www.editorialmanager.com/pone/login.asp?a=r)*. Please contact the publication office if you have any questions.*
