# Supplementary material for: Demographic, social and health system factors associated with maternal mortality in Pakistan: A nested case-control study
Source: PLoS One. 2025 Jan 3;20(1):e0290492. doi: 10.1371/journal.pone.0290492 (PMC11698442; doi:10.1371/journal.pone.0290492)
Supplement: Attachment — Submitted filename: Response to Reviewers_Oct 13th 2024.docx [file pone.0290492.s005.docx]

**Response to Reviewers**

Note: Responses are show in *Italic*.

Journal Requirements:

Please review your reference list to ensure that it is complete and correct. If you have cited papers that have been retracted, please include the rationale for doing so in the manuscript text or remove these references and replace them with relevant current references. Any changes to the reference list should be mentioned in the rebuttal letter that accompanies your revised manuscript. If you need to cite a retracted article, indicate the article’s retracted status in the References list and also include a citation and full reference for the retraction notice.

***Response:*** *We have reviewed reference list for completeness and correctness. Changes have been made to correct the title or other information. No retracted paper was (and is) in the reference list.*

[Note: HTML markup is below. Please do not edit.]

Reviewers' comments:

Reviewer's Responses to Questions

**Comments to the Author**

1. If the authors have adequately addressed your comments raised in a previous round of review and you feel that this manuscript is now acceptable for publication, you may indicate that here to bypass the “Comments to the Author” section, enter your conflict of interest statement in the “Confidential to Editor” section, and submit your "Accept" recommendation.

Reviewer #2: All comments have been addressed

Reviewer #4: (No Response)

***Response:*** *Thank you.*

2. Is the manuscript technically sound, and do the data support the conclusions?

The manuscript must describe a technically sound piece of scientific research with data that supports the conclusions. Experiments must have been conducted rigorously, with appropriate controls, replication, and sample sizes. The conclusions must be drawn appropriately based on the data presented.

Reviewer #2: Yes

Reviewer #4: Yes

***Response:*** *Thank you.*

3. Has the statistical analysis been performed appropriately and rigorously?

Reviewer #2: Yes

Reviewer #4: (No Response)

***Response:*** *Thank you.*

4. Have the authors made all data underlying the findings in their manuscript fully available?

The [PLOS Data policy](http://c05y1x9s.r.us-east-2.awstrack.me/L0/http:%2F%2Fwww.plosone.org%2Fstatic%2Fpolicies.action%23sharing/1/010f01926c8902bf-5927bb16-c142-4b25-ba06-a69dc4683d2a-000000/9JHW0wTU5JaXS1-rBe42F_ZIbf8=179) requires authors to make all data underlying the findings described in their manuscript fully available without restriction, with rare exception (please refer to the Data Availability Statement in the manuscript PDF file). The data should be provided as part of the manuscript or its supporting information, or deposited to a public repository. For example, in addition to summary statistics, the data points behind means, medians and variance measures should be available. If there are restrictions on publicly sharing data—e.g. participant privacy or use of data from a third party—those must be specified.

Reviewer #2: Yes

Reviewer #4: Yes

***Response:*** *Thank you.*

5. Is the manuscript presented in an intelligible fashion and written in standard English?

PLOS ONE does not copyedit accepted manuscripts, so the language in submitted articles must be clear, correct, and unambiguous. Any typographical or grammatical errors should be corrected at revision, so please note any specific errors here.

Reviewer #2: Yes

Reviewer #4: Yes

***Response:*** *Thank you.*

6. Review Comments to the Author

Please use the space provided to explain your answers to the questions above. You may also include additional comments for the author, including concerns about dual publication, research ethics, or publication ethics. (Please upload your review as an attachment if it exceeds 20,000 characters)

Reviewer #2: All Comments had been addressed.

***Response:*** *Thank you.*

All the best

Reviewer #4: Dear Authors,

It is good Job. The study used nested case-control design with robust statistical analysis to better understand maternal mortality in Pakistan. I have some comments that can improve your work.

***Response:*** *Thank you.*

- what does your "main findings mean" merely should be stated in abstract section of the conclusion.

***Response:*** *Thank you. We have now included the main findings under Conclusions in the Abstract. Specifically, we have added: “This study identifies secondary or higher maternal education, having had tetanus injection during the last pregnancy, ever-used contraception or being in the age group of 20-29 years were factors associated with lower risk of maternal mortality. Conversely, skilled birth attendance increases the risk of maternal death in Pakistan”.*

- The study shows sigificant association between SBAs and high MMR. Could this works for causality too? or How can the authors address limitations of drawing conclusions on the causality?

***Response*:** *Thank you for raising an important point. Out data and statistical technique of case-control analysis do not permit drawing causal relationship between any predictor, including delivery by skil bi9rth attendants. While not drawing causality, we discuss the relationship between delivery by skill birth attendant and risk of maternal death.*

*Lines 266-288 of the manuscript discuss the association between SBAs and risk of maternal mortality as follows: “However, the finding that women whose last delivery was by a skilled birth attendant had higher odds of maternal death is counterintuitive, though consistent in both PDHS 2006-07 [8] and our analysis of PMMS data. There are three explanations for this unexpected result derived from the in-depth analysis of the verbal autopsies of the deceased women identified in the PMMS [9]. First, women after having sought care from diverse types of care providers for symptoms/complications of pregnancy reached the skilled attendant with a severe complication/symptom(s) in a critical condition in many cases after shuffling between two to three facilities before reaching the final referral facility. The in-depth analysis further showed that most maternal deaths occurred at health facilities following delays in deciding to seek professional care and in reaching an appropriate health facility for care [9].In a social-cultural context with little or no birth planning and accessing care when situation is worsened, this selectivity in terms of women with a complicated pregnancy or delivery reaching late a skilled attendant, manifested in a higher risk of death. Also, women with prior history of symptoms or those requiring cesarean section are more likely to approach skilled attendant for delivery. Such a pattern is noted where an effective referral system is not operational. A study of rural areas of Balochistan and North-West Frontier Province (now renamed as Khyber Pakhtunkhwa) showed a similar pattern of elevated risk of maternal mortality for women who had delivered by a skilled attendant compared to those by a family member or traditional birth attendant [11]. A similar pattern was also observed in eight urban squatter settlements of Karachi [12].*

*Second, it was found that skilled attendants were not adequately trained and provided a sub-optimal quality of care. The in-depth analysis shows that 36% of all maternal deaths, direct or indirect, and 91% of direct maternal deaths were due to surgical or medical misadventures [9]. Indifference by unmotivated staff, poor skills of health care providers, and lack of medicine and equipment together contributed to heavy death toll of women reaching the facility in reasonable condition [9]. Third, the finding of higher maternal mortality among women with births attended by a skilled provider is consistently reported in studies from Pakistan and the qualitative analysis of verbal autopsies indicates that women with complicated pregnancy or delivery to start with were more likely to seek care from a skilled attendant. The pathway to skilled delivery is, therefore, shaped by the prior history of symptoms exacerbating pregnancy complications leading to institutional care and delivery by a skilled attendant. In addition, the poor quality of service, including poor skills of providers contribute to higher maternal mortality for women with delivery by a skilled attendant. This noteworthy finding indicates that the emphasis on institutional deliveries and delivery by a skilled birth attendant to reduce maternal mortality is insufficient to reduce maternal mortality where norms for pregnancy care are lacking, but a necessary concomitant of complicated pregnancies. Moreover, it is imperative to ensure the quality of care and enhance the skills of health care providers to effectively manage the serious complications of women reaching the facility.”*

*We discuss limitations of the study in lines 266-288 of the manuscript.*

- Why pakistan still have high number of MMR despite considerable improvements.

***Response:*** *Thank you for the thought-provoking question. Despite considerable improvement, Pakistan continues to experience high level of maternal mortality as compared to other countries in South Asia. One of the main reason is the growing number of women entering reproductive age while contraceptive use has been stagnated and high number of pregnancies continue to occur many of them unintended. Pregnancies of young women and unintended pregnancies are of high risk of morbidity and mortality. We discuss this aspect in Lines 336-361 with additional text in Lines 359-361 as follows: “Third, family planning should be promoted for maternal and child survival through birth spacing and prevention of unintended and high-risk pregnancies. According to the PDHS 2017-18, nearly 18% of women became pregnant six to 17 months after a live birth and 37% within 24 months. PDHS data show that the highest proportion of closely spaced pregnancies occur in the adolescent age group of 15-19 years. Compared to older women, this group also has the highest unmet need for birth spacing—one out of six women in this age bracket wants to space their pregnancies but is unable to do so.*

*Unmet need for family planning (FP) stems from the inability of women to access services. The Lady Health Worker (LHW) program, launched in 1994, had the mandate to provide doorstep FP services to rural women, who have higher unmet need and more unintended pregnancies than urban women. This highly acclaimed program is currently plagued with many issues, the foremost of which is a perennial shortage of contraceptive supplies. Within public health facilities, service providers do not consider offering family planning services and counselling as their responsibility. This needs to be changed through some bold decisions by the government. A major step should be mandatory provision of FP services through the public and private health networks. This has several advantages, including avoiding the hesitancy some women and men have in visiting the socially stigmatized Family Planning centers run by the Population Welfare Department. General health facility visits offer several opportunities for discussing family planning, such as during antenatal care visits, immediately after delivery, during postnatal checkups, and child immunization visits. On these occasions, couples can be encouraged to discuss their family planning needs and individually focused options can be suggested to help meet their needs. This service delivery approach would also allow men to discuss family planning with male health care providers.*

*The Population Council estimates that even without increasing the current coverage of skilled birth attendance; by simply meeting the 17% unmet need for family planning and thus raising current contraceptive use from 34% to 51%, Pakistan could lower maternal mortality by around 30% and save every year 4,000 maternal lives. With increasing number of women continuing to enter reproductive ages without little or no concomitant rise in modern contraceptive use, women will continue to experience high number of pregnancies that expose them to risks of maternal morbidity and mortality”.*

- I see some of the section of the references are not full. Please re consider it

***Response:*** *Thank you for the comment. We have reviewed and checked references for completeness and correctness.*

7. PLOS authors have the option to publish the peer review history of their article ([what does this mean?](https://c05y1x9s.r.us-east-2.awstrack.me/L0/https:%2F%2Fjournals.plos.org%2Fplosone%2Fs%2Feditorial-and-peer-review-process%23loc-peer-review-history/1/010f01926c8902bf-5927bb16-c142-4b25-ba06-a69dc4683d2a-000000/nOecbr7OqlA56skA-3wcDWzPOsk=179)). If published, this will include your full peer review and any attached files.


If you choose “no”, your identity will remain anonymous but your review may still be made public.


**Do you want your identity to be public for this peer review?** For information about this choice, including consent withdrawal, please see our [Privacy Policy](https://c05y1x9s.r.us-east-2.awstrack.me/L0/https:%2F%2Fwww.plos.org%2Fprivacy-policy/1/010f01926c8902bf-5927bb16-c142-4b25-ba06-a69dc4683d2a-000000/8c0DubL9jn0ffivXgH2TRMd6UXg=179).

Reviewer #2: **Yes:** Dr. Zalikha Khamis Al-Marzouqi

Reviewer #4: **Yes:** Eskinder Israel

[NOTE: If reviewer comments were submitted as an attachment file, they will be attached to this email and accessible via the submission site. Please log into your account, locate the manuscript record, and check for the action link "View Attachments". If this link does not appear, there are no attachment files.]

While revising your submission, please upload your figure files to the Preflight Analysis and Conversion Engine (PACE) digital diagnostic tool, [https://pacev2.apexcovantage.com/](https://c05y1x9s.r.us-east-2.awstrack.me/L0/https:%2F%2Fpacev2.apexcovantage.com%2F/1/010f01926c8902bf-5927bb16-c142-4b25-ba06-a69dc4683d2a-000000/jn_czWx-0V8lJJsyMv9lC36IcK4=179). PACE helps ensure that figures meet PLOS requirements. To use PACE, you must first register as a user. Registration is free. Then, login and navigate to the UPLOAD tab, where you will find detailed instructions on how to use the tool. If you encounter any issues or have any questions when using PACE, please email PLOS at [figures@plos.org](mailto:figures@plos.org). Please note that Supporting Information files do not need this step.

*In compliance with data protection regulations, you may request that we remove your personal registration details at any time.* [*(Remove my information/details)*](https://c05y1x9s.r.us-east-2.awstrack.me/L0/https:%2F%2Fwww.editorialmanager.com%2Fpone%2Flogin.asp%3Fa=r/1/010f01926c8902bf-5927bb16-c142-4b25-ba06-a69dc4683d2a-000000/Aht4B3064XHGJAEGjcDonl0Tqec=179)*. Please contact the publication office if you have any questions.*
